# Effects of stage I hypertension on the recovery of early postoperative attention network function in elderly patients undergoing elective hip or knee arthroplasty surgery

**DOI:** 10.3906/sag-1902-58

**Published:** 2020-02-13

**Authors:** Dan ZHAO, Jun LI, Rui YANG, Guang-hong XU

**Affiliations:** 1 Department of Anesthesiology, First Affiliated Hospital, Anhui Medical University, Hefei, Anhui P.R. China; 2 Department of Neurology, First Affiliated Hospital, Anhui Medical University, Hefei, Anhui P.R. China

**Keywords:** Hypertension, attentional network test, alerting, orienting, executive control, general anesthesia

## Abstract

**Background/aim:**

Hypertension is an important risk factor for cognitive impairment. This study explored whether elderly patients with stage I hypertension (HPs) and normotensive patients (NPs) showed differences in the recovery of postoperative attention network function according to the attentional network test (ANT) performance.

**Materials and methods:**

Of 110 patients screened, 25 HPs and 25 NPs completed this study. The Mini-Mental State Examination (MMSE) was applied to all participants before the operation and the ANT (on days 2 and 7) after the operation. All participants completed 1 day preoperatively and the ANT on postoperative days (PODs) 2 and 7.

**Results:**

Compared with NPs, HPs had significantly lower alerting network effect scores and more difficulty resolving conflict on POD 7. However, no significant difference was observed between the groups on POD 2. Orienting network performance was similar between the groups at all time points. Significant differences in alerting and executive control network performances were observed between PODs 2 and 7 in each group.

**Conclusion:**

HPs showed selective cognitive impairment at different time points following elective hip or knee arthroplasty. Compared with NPs, during the first postoperative week, HPs were more likely to experience delayed recovery of alerting and executive control network function, but not orienting network function.

## 1. Introduction

Loss of cognitive function is one of the most devastating manifestations of aging and vascular disease. Chronic hypertension has been associated with increased risks of cognitive decline, vascular dementia, and Alzheimer disease [1], and is regarded as a key modifiable risk factor for age-related dementia [2]. Previous studies have shown that antihypertensive treatment can improve cognitive performance and prevent dementia in elderly people [3,4]. However, other studies have shown that low blood pressure (BP) contributes to brain atrophy and more rapid cognitive impairment in hypertensive patients with no apparent cerebrovascular disease [3,5]. These contradictory effects may be explained by differences in antihypertensive agents used, patient characteristics, and the extent of blood pressure reduction [3].

Postoperative cognitive dysfunction (POCD) is a common complication occurring after surgery and anesthesia. It affects patients in all age groups, including young and middle-aged patients, but particularly impacts elderly patients (age > 60 years) [6]. POCD has been associated with various durations and types of surgery, the use of certain anesthetics, and hypertension [7]. It affects diverse cognitive domains, including memory, attention, and executive function [8,9].

The term “attention” encompasses several psychological phenomena [10], and the human attentional system has been divided into three independent networks: alerting, orienting, and executive control. The attentional network test (ANT) has been used widely to examine these attentional functions in healthy subjects and patients with different neuropsychiatric disorders including schizophrenia and Alzheimer disease [11,12]. In this study, we used the ANT to evaluate whether there is a difference in attention functions of elderly patients with hypertensive and normotensive in the postoperative period, and to further examine the types of cognitive change and the recovery of cognitive function in the patients with hypertension after surgery and anesthesia. We aimed to test whether or not hypertension is an important risk factor for postoperative cognitive impairment, which would present as a postoperative attentional network impairment [13,14], in elderly patients. Therefore, we compared attentional network functions in elderly patients with normotension and hypertension after surgery. 

## 2. Materials and methods

### 2.1. Participants and ethical approval

Totally 110 patients with stage I hypertension (HPs) and normotensive patients (NPs) undergoing elective hip or knee arthroplasty were screened and 50 patients (age range: 60–80 years) completed the study. Subjects with self-reported histories of essential hypertension (lasting 3–5 years) and systolic BPs of 140–159 mmHg or diastolic BPs of 90–99 mmHg were assigned to the HP group. Those with systolic BPs of 90–140 mmHg or diastolic BPs of 60–90 mmHg were included in the NP group. The BP was assessed again and patients’ charts were reviewed 1–3 days preoperatively. A cardiologist reviewed all participants’ medical records and determined their hypertension status using the diagnostic criteria of the American Heart Association [15].

**Figure 1:**
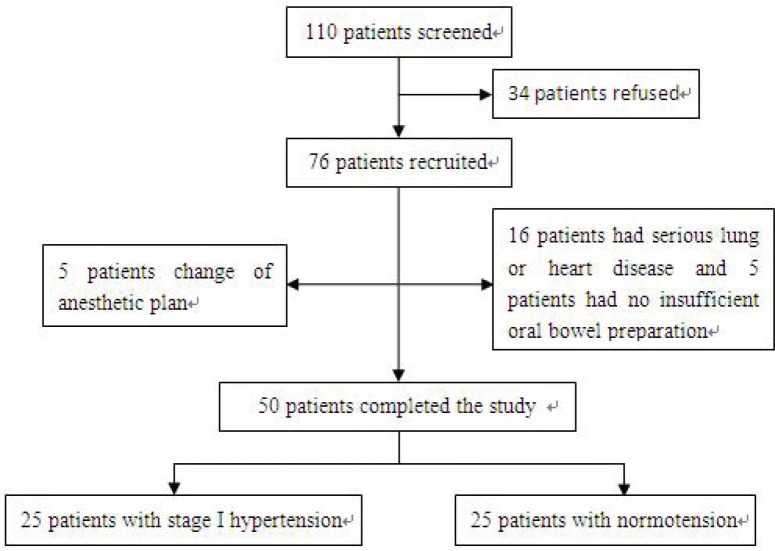
Flowchart showing details of clinical procedures throughout the study.

Study exclusion criteria were: American Society of Anesthesiologists (ASA) physical status III or IV, secondary hypertension (e.g., tumor or renal disease), history of smoking or alcohol abuse, dyslipidemia or obesity (body mass index ≥ 25 kg/m2), inadequate comprehension of Chinese language, history of psychiatric or neurological disorder, diabetes mellitus, and cerebrovascular lesion (e.g., subdural hematoma). Patients with abnormal Mini-Mental State Examination (MMSE) results for their levels of education (e.g., MMSE score < 22 for a patient with no education) and those unwilling to comply with the protocol or procedures at any time during the study were excluded. Patients who had received regular antihypertensive drug treatment previously or had histories of low BP were also excluded. A flow chart summarizing patient enrollment and exclusion criteria is shown in Figure 1.

This study was approved by the Ethics Committee of Anhui Medical University, Hefei, Anhui, China. The patients were included in the study after providing written informed consent, and all procedures were conducted according to the Declaration of Helsinki.

### 2.2. Anesthesia

In accordance with our standard procedure, general anesthesia was induced with etomidate (0.15–0.3 mg/kg) and midazolam (0.01–0.05 mg/kg) in combination with sufentanil (0.2–0.5 μg/kg), and followed by neuromuscular blockade with cisatracurium (0.2–0.3 mg/kg) to facilitate laryngeal mask airway insertion. Propofol [target controlled infusion (1–4 μg/mL) or constant rate infusion (3–6 mg/kg)], remifentanil (0.1–0.4 μg/kg/min), and intermittent cisatracurium bromide were used to maintain the appropriate depth of anesthesia. Routine monitoring included electrocardiography and the measurement of BP, oxygen saturation, end-tidal carbon dioxide concentration, and heart rate. In addition, bispectral index (BIS) monitoring (Vista, Aspect Medical System Inc., USA) was used to determine the depth of anesthesia for all the patients. All the patients received local infiltration of 0.5% ropivacaine and flurbiprofen axetil for pain relief at the end of surgery.

### 2.3. Attentional network test (ANT)

**Figure 2:**
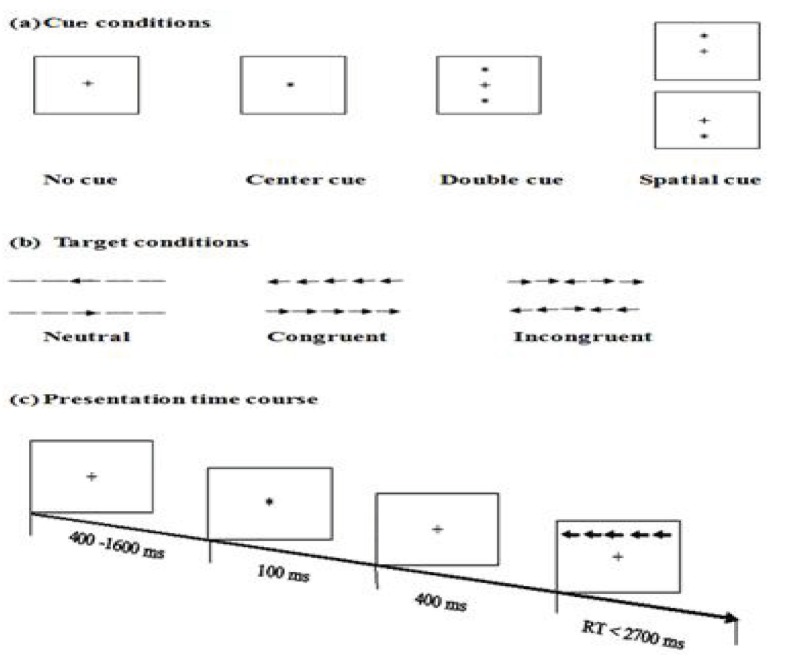
Experimental paradigm of the ANT. (a) The four cue conditions; (b) six stimuli used in the present experiment; and (c) an example of the procedure.

The ANT used in this study was created by using E-Prime (version 1.1, Psychology Software Tools, Pittsburgh, PA, USA). Stimuli were presented on a 17-inch color monitor controlled by a personal computer. Participants viewed each stimulus and responded by pressing one of two keys (“←” or “→”) with the left or right index finger, respectively. Each stimulus was presented with a row of five horizontal black lines with arrowheads pointing left or right. The target was a left- or right-pointing arrowhead at the center of the field against a gray background, flanked on either side by two arrows pointing in the same direction (congruent condition) or the opposite direction (incongruent condition), or by nothing (neutral condition; Figure 2).

The arrow appeared below or above a fixation point (“+”), preceded or not by a warning cue (“*”), and accompanied or not by flankers (two arrows on either side). The participants were requested to focus on a centrally located stationary cross throughout the task and to respond as quickly and accurately as possible by pressing the key corresponding to each target arrow’s direction. The first fixation duration varied randomly (400–1600 ms). The second fixation, presented with or without a warning cue, was 100 ms in duration. Four hundred milliseconds after cue offset, the target and flankers appeared simultaneously and remained on the screen until the participant responded with a button press (or for a maximum of 2700 ms).

The ANT was performed with four cue conditions: (1) no cue, in which a cross appeared in the same location as the first stationary cross for 100 ms; (2) center cue, in which an asterisk was presented at the center of the monitor; (3) double cue, in which asterisks were presented at two target locations (above and below the central point) simultaneously; and (4) spatial cue, in which one asterisk was presented at a target location above or below the central point (Figure 2).

In accordance with the ANT design principles, the experiment included a 24-trial practice block and three experimental trial blocks randomly ordered. Each experimental block consisted of 96 trials (48 conditions: four cue types × two target locations × two target directions × three congruencies, with two repetitions). The entire ANT was completed in 30 min. Both patients and postoperative observers were blinded to group allocation.

### 2.4. Calculation of attentional network efficiency

The ANT uses differences in response times (RTs) derived from network-specific experimental conditions to measure the alerting, orienting, and executive control networks [16]. Alerting network effect scores were calculated by subtracting the mean RTs from double-cue trials from those from no-cue trials, with higher scores indicating more readiness to respond to an impending stimulus. Orienting network effect scores were calculated by subtracting the mean RTs from spatial-cue trials from those from center-cue trials, with higher scores reflecting faster covert orienting of attention to a spatially cued location. Executive control network effect scores were calculated by subtracting the mean RTs of congruent-target trials from RTs of incongruent-target trials. A higher execution effect score indicated more severe execution dysfunction because a longer time was needed to execute correctly upon the incongruent cue, and there were more difficulties in resolving conflict.

### 2.5. Statistical analysis

Sex, age, education level, duration and type of surgery, MMSE score, and the three attention network scores were compared between the groups using independent-sample t-tests and chi-square tests. We used repeated-measures analysis of variance to evaluate the differences in the three attention network scores, with timing [postoperative days (PODs) 2 and 7] serving as the within-subject factor and group (HP and NP) serving as the between-subject factor. All analyses were conducted with SPSS software (ver. 13.0, SPSS Inc., Chicago, IL, USA). Two-sided P-values of <0.05 were considered to be significant.

## 3. Results

Of the 110 patients screened in this study, 9 male and 7 female patients were excluded because of serious lung or heart disease (ASA physical status III or IV), 3 male and 2 female patients were excluded because of insufficient oral bowel preparation, 2 male and 3 female patients were excluded due to the alteration of the anesthetic plans (epidural anesthesia), and 16 male and 18 female patients declined to participate postoperatively. A total of 50 patients (25 HPs and 25 NPs) completed the study. Patient characteristics, duration and type of surgery, blood loss, transfusion volume, and propofol, midazolam, remifentanil, and sufentanil doses did not differ significantly between the groups (Table 1).

**Table 1:** Demographic characteristics and intraoperative data.

Characteristic	Hypertensive group	Normotensive group	P-value	χ2 OR t value
Age, years	65.80 ± 4.65	64.56 ± 3.84	0.31	0.91
Sex (M/F)	13/12	12/13	0.78	0.08
Weight, kg	67.60 ± 9.97	65.92 ± 12.58	0.60	0.53
MMSE	23.60 ± 3.39	23.44 ± 3.27	0.86	0.21
Educational level (years)	6.48 ± 5.52	5.13 ± 5.3	0.39	0.86
Illiteracy (not educated), n (%)	10 (40.00)	11 (44.00)		
Primary school, n (%)	7 (28.00)	6 (24.00)		
Junior middle school, n (%)	2 (8.00)	3 (12.00)		
Senior middle school, n (%)	2 (8.00)	3 (12.00)		
University, n (%)	4 (16.00)	2 (8.00)		
ASA class			0.77	0.08
I, n (%)	12 (48.00)	13 (52.00)		
II, n (%)	13 (52.00)	12 (48.00)		
Duration of surgery, min	113.72 ± 28.18	110.00 ± 16.89	0.57	0.56
Intraoperative bleeding, mL	490.00 ± 210.16	500.00 ± 129.10	0.84	–0.19
Intraoperative fluid infusion, mL	2080.0 ± 471.7	2272.0 ± 566.1	0.19	–1.3
Midazolam dose, mg	1.64 ± 0.2	1.74 ± 0.2	0.1	–1.6
Propofol dose, mg	436.80 ± 103.44	480.80 ± 78.10	0.09	–1.7
Remifentanil dose, mg	1.42 ± 0.32	1.60 ± 0.36	0.07	–1.8
Sufentanil dose, µg	45.50 ± 7.47	47.80 ± 4.10	0.18	–1.3
Type of surgery			0.57	0.3
Hip arthroplasty surgery, n (%)	11 ( 44.00)	13 (52.00 )		
Knee arthroplasty surgery, n (%)	14 (56.00)	12 (48.00)		

Data are presented as mean ± standard deviation or n (%). *P < 0.05. MMSE= Mini-Mental State Examination; ASA= American Society of Anesthesiologists.

### 3.1. Accuracy

In this study, accuracy was measured as the percentage of overall correct responses in the ANT trials. The overall mean RTs and accuracy values for the ANT did not differ significantly between the groups for any of the three attentional networks at any time point (Table 2).

**Table 2:** Attentional network test response times and accuracy on postoperative days 2 and 7.

		Normotensive group			Hypertension group	
Item	2 DPO	7 DPO	P-value	2 DPO	7 DPO	P-value
Alerting (ms)	36.84 ± 15.41	62.64 ± 34.82	0.001*	28.48 ± 23.52	40.44 ± 23.21	0.002*
Orienting (ms)	29.80 ± 26.52	36.52 ± 39.32	0.753	30.88 ± 15.62	38.24 ± 24.54	0.059
Executive (ms)	87.52 ± 27.16	48.48 ± 36.65	<0.001*	83.00 ± 34.36	69.20 ± 26.48	0.009*
Accuracy (%)	96.32 ± 4.43	96.84 ± 3.04	0.903	98.24 ± 2.31	97.36 ± 2.27	0.179
Overall mean RT	1019.9 ± 172.5	905.6 ± 255.2	0.076	1030.0 ± 129.7	1001.4 ± 177.3	0.394

Data are presented as the mean ± standard deviation. *P < 0.05, **P < 0.01. POD= postoperative day; RT= response time.

### 3.2. Attentional network effect scores

Effect scores did not significantly differ between the groups on POD 2 (Table 3). However, effect scores for the alerting and executive control network tasks differed between the groups on POD 7 and within each group between PODs 2 and 7 (Tables 2 and 3). Compared with NPs, HPs had significantly lower alerting network effect scores and more difficulty in resolving conflict on POD 7. No significant difference in the score for the orienting network task was observed between groups at any time point (Tables 2 and 3). Both groups had significantly lower alerting network effect scores and more difficulty in resolving conflict on POD 2 than on POD 7, with no significant difference for the orienting network task.

**Table 3:** Attentional network test response times and accuracy on postoperative days 2 and 7.

Item	Normotensive group	Hypertension group		Normotensive group	Hypertension group		2 DPO	2 DPO	P-value	7 DPO	7 DPO	P-value
	Alerting (ms)	36.84 ± 15.41	28.48 ± 23.52	0.144	62.64 ± 34.82	40.44 ± 23.21	0.011*
Orienting (ms)	29.80 ± 26.52	30.88 ± 15.62	0.871	36.52 ± 39.32	38.24 ± 24.54	0.212
Executive (ms)	87.52 ± 27.16	83.00 ± 34.36	0.608	48.48 ± 36.65	69.20 ± 26.48	0.026*
Accuracy (%)	96.32 ± 4.43	98.24 ± 2.31	0.610	96.84 ± 3.04	97.36 ± 2.27	0.830
Overall mean RT	1019.9 ± 172.5	1030.0 ± 129.7	0.816	905.6 ± 255.2	1001.4 ± 177.3	0.130

Data are presented as the mean ± standard deviation. *P < 0.05. POD= postoperative day; RT= response time.

## 4. Discussion

Attention is an umbrella term for various psychological phenomena [10]. Posner divided the human attentional system into three independent networks: alerting, orienting, and executive control. These networks have been distinguished at the biochemical and cognitive levels, and they have been confirmed to have distinct neuroanatomical correlates [17,18]. The ANT is based on the flanker and exogenous cueing paradigms, measures the activities of all three networks simultaneously, and evaluates their interrelationships [16], which has been used widely to examine attentional functions in both healthy individuals and patients with various diseases [11,12]. In the current study, we used the ANT test to evaluate the difference in attention functions of patients with hypertension and normotension in the postoperative period, and to further examine the types of cognitive change and the recovery of cognitive function in hypertensive patients after surgery and anesthesia. We found no significant difference in the function of the three attentional networks in the ANT test between the groups on POD 2, but reduced alerting and executive control network function in HPs on POD 7. Elderly HPs undergoing elective hip or knee arthroplasty displayed significant cognitive impairment on POD 7 relative to NPs on POD 7 and to both groups on POD 2. Taken together, the two groups had similar cognitive impairment (affecting the alerting and executive control networks) on POD 2 and obvious cognitive improvement on POD 7. However, the findings indicate that stage I hypertension can delay the rate of cognitive recovery in elderly patients undergoing surgery with anesthesia.

Cognitive decline is an important cause of disability and mortality [19], and growing evidence suggests that hypertension is a risk factor for cognitive decline [1]. However, the relationship between hypertension and cognition has not been clarified and may be complex. Particular cognitive functions involve specific brain regions (e.g., the medial temporal lobe for memory). Disruption of any brain system during the perioperative period has the potential to induce cognitive changes. The alerting network, which is localized to the thalamus and the right frontal and parietal areas, is responsible for the activation and maintenance of a vigilant state [20]. Executive network function involves the frontal areas and can be impaired by lesions on the prefrontal cortex [21]. Because HPs had reduced alerting network effect scores and greater difficulty resolving conflict than NPs did on POD 7 in this study, we suspect that they sustained more substantial damage to the frontal and parietal areas of the brain. Magnetic resonance imaging (MRI) studies have revealed altered patterns involving the parietal and frontal lobes in HPs relative to NPs [22]. The frontoparietal network is important for executive function, attention control, and working-memory processing [23]. Thus, the impairments in executive functioning and alerting exhibited by HPs in this study could be related to damaged functional connectivity of the frontal and parietal regions. HPs have shown deficits in executive function, memory, and attention [24]. In a recent perfusion MRI study, Hajjar et al. [25] found that hypertensive patients had blunted responses to carbon dioxide in the frontal and parietal regions, which correlated with poor outcomes in microvascular ischemic injury and macrovascular brain disease. Dysfunctional connectivity in the frontal and parietal regions may be related to hypertension pathology; if so, it may reflect the neural mechanism mediating the increased risk of cognitive deficits, including POCD, in HPs. Our findings provide new insight on the mechanisms of cognitive dysfunction and the recovery of cognitive function in HPs after surgery and anesthesia.

In the current study, we found no significant difference between the groups in the performance of the orienting network at any time point, suggesting that this network is less sensitive than the other two attention networks. Although hypertension may eventually affect all of these domains, executive function is commonly affected earlier [26]. In a previous study, HPs showed declines in mainly executive function and attention compared with NPs, possibly due to the impact of white matter on executive function mediated by the frontoparietal network in the former [22]. In this study, HPs manifested mainly executive function impairment supporting this evidence indirectly, without orienting network decline.

One potential limitation of the current study was the lack of brain imaging, which may have allowed us to identify injured brain regions. In addition, we administered only the MMSE, with no ANT, at baseline. The MMSE is still a widely used and accepted cognitive assessment scale. Our results showed no significant difference between the groups in the three attentional networks on POD 2, but significant differences in the type and severity of ANT between the groups on POD 7, indicating that surgery and anesthesia can induce cognitive impairment in elderly patients undergoing elective hip or knee arthroplasty. However, compared with NPs, HPs may experience delayed postoperative recovery cognition. In addition, the long-term effects of hypertension on postoperative cognition were not examined in this study. In future studies, we will examine the impacts of antihypertensive drugs and hypertension type (systolic versus diastolic) on postoperative cognition.

In summary, HPs undergoing elective hip or knee arthroplasty appeared to have selective cognitive impairment at different postoperative time points. Compared with NPs, HPs were more likely to experience delayed recovery of alerting and executive control network function. The orienting network was not affected. Functional disconnection of the frontal and parietal regions may play an important role in POCD in HPs. We believe that the monitoring of cognitive function for hypertension is necessary after anesthesia and surgery.

## Acknowledgments

We would like to thank Drs. Qin Zhao, Xin-qi Cheng, and Hu Liu for their assistance with this study. This work was funded by the National Nature Science Foundation of China (Grant No. 81870837) and the Natural Science Research Project of the Anhui Institution of Higher Learning, Anhui Provincial Education Department, China, No. KJ2018A0189.
